# Association between levels of tumor-infiltrating lymphocytes in different subtypes of primary breast tumors and prognostic outcomes: a meta-analysis

**DOI:** 10.1186/s12905-020-01038-x

**Published:** 2020-09-05

**Authors:** Lin He, Yaling Wang, Qian Wu, Yuhua Song, Xuezhen Ma, Biyuan Zhang, Haiji Wang, Yong Huang

**Affiliations:** 1 Breast Disease Center, The Affiliated Hospital of Qingdao University, Qingdao, Shandong Province People’s Republic of China; 2 Department of Oncology, The Second Affiliated Hospital of Medical College of Qingdao University, Qingdao, Shandong Province People’s Republic of China; 3 Department of Radiotherapy, The Affiliated Hospital of Qingdao University, Qingdao, Shandong Province People’s Republic of China

**Keywords:** Breast cancer, tumor-infiltrating lymphocytes, Overall survival, Pathological complete response, Meta-analysis

## Abstract

**Background:**

To investigate the impact of the elevation of tumor-infiltrating lymphocytes (TILs) in different molecular subtypes of primary breast cancer, i.e. each 10% increment of TILs and high-level TILs (TILs≥50%) in tumor, on overall survival (OS) and pathological complete response (pCR) and to compare the presentation of high-level TILs across these molecular subtypes.

**Methods:**

Citation retrieval was performed in the PubMed, Cochrane Library, Embase and Web of Science databases. All statistical calculations were performed by the software of StataSE version 12.0.

**Results:**

Twenty-two eligible clinical trials including 15,676 unique patients were included for meta-analysis. Each 10% increment of TILs significantly improved OS in human epidermal growth factor receptor 2 (HER2)-overexpression (pooled Hazard ratio (HR), 0.92; 95% CI, 0.89–0.95) and triple-negative (TN) (pooled HR, 0.90; 95% CI, 0.89–0.92) breast tumors but not in luminal tumor subtype (pooled HR, 1.06; 95% CI, 0.99–1.13). It was also associated with an increased pCR rate in breast cancers (pooled Odds ratio (OR), 1.27; 95% CI, 1.19–13.5). High-level TILs were significantly related with a higher pCR rate (pooled OR, 2.73; 95% CI, 2.40–3.01) than low-level TILs. The HER2-amplified (pooled OR, 3.14; 95% CI, 1.95–5.06) and TN (pooled OR, 4.09; 95% CI, 2.71–6.19) phenotypes of breast cancers expressed significantly more high-level TILs than the luminal tumor subtype, although the presentation of those between the former two subsets was not significantly different (pooled OR, 1.30; 95%CI, 0.83–2.04).

**Conclusions:**

The elevation of TILs in breast tumors predicts favorable prognostic outcomes, particularly in the HER2-overexpression and TN subtypes.

## Background

Tumor microenvironment is thought to play an important role in the germination, development, invasion and metastasis of tumors and is composed of immune cells, cytokines, adipocytes, and cancer-related fibroblasts, as well as the extracellular stroma [1, 2]. The interaction of immune lymphocytes and tumor cells is cardinal in these procedures. In the immune system, lymphocytes can eradicate tumor cells and prevent neoplasm development through immune surveillance [3]; tumor-infiltrating lymphocytes (TILs) participate in the regulation of the tumor niche and the inhibition of tumor formation and development [2].

High-level TILs favor a good, long-term prognosis and enhanced chemosensitivity in primary aggressive molecular subtypes of breast cancer, including the human epidermal growth factor receptor 2 (HER2)-positive (HER2/neu oncogene overexpressed, estrogen receptor (ER)-negative) and triple-negative (TN) subtypes. When TN breast cancer patients undergo chemotherapy, each 10% increment of intratumoral TILs (iTILs) and stromal TILs (sTILs) leads to reductions of the recurrence risk of 17 and 15%, respectively, and to reductions of the death risk of 27 and 17%, respectively [4]. The presentation of high-level TILs is also positively associated with the survival benefits of anthracycline-based chemotherapy and anti-HER2 targeted therapy (trastuzumab) in HER2-positive breast tumors [5]. Of note, a pooled analysis of 3371 patients who underwent neoadjuvant therapy had a higher concentration of TILs, which led to a shorter overall survival (OS) than lower concentrations in the luminal phenotype of breast cancer, [6] suggesting a different biological feature of immune infiltration in this tumor subtype.

In this context, the purpose of our study is to settle these issues, including how each 10% increment of TILs and high-level TILs in breast cancer and in three tumor phenotypes (luminal, HER2-overexpression and TN) influence the OS and the pathological complete response (pCR) rate. We also compare the expression of high-level TILs across these molecular subsets.

## Methods

### Search strategy

Electronic retrievals were performed from the PubMed, Web of Science, Cochrane Library and Embase databases according to the following search strategy: ((primary breast cancer) OR (primary breast tumor) OR (primary breast tumor)) AND ((tumor-infiltrating lymphocytes) OR (immune cells infiltration) OR (immune cells infiltrating) OR (immune cell infiltration) OR (immune cell infiltrating)) NOT (metastasis OR metastatic OR metastasize). No restrictions were used during the retrieval process. The deadline for retrieval was 25 March 2019.

### Inclusion criteria

- Clinical trials;
- Female patients with primary a breast tumor;
- The impact of each 10% increment of TILs or high-level TILs in breast cancer on the OS or on the pCR rate was reported in publications. Studies that documented at least two molecular tumor subtypes with the expression of high-level TILs were also included. TILs were quantified on hematoxylin and eosin–stained sections and evaluated by the usage of the guideline of the International TILs Working Group [7]. OS referred to the duration from the date of diagnosis to the date of death or lost follow-up. pCR was defined as the pathologically absent residual tumor foci in the breast and local regional lymph nodes. The definition of the high-level TILs was the TIL’s concentration in breast tumors greater than 50%.

### Exclusion criteria

- Articles not published in English;
- Studies referencing forkhead box P3 (FOXP3) + or programmed death 1 (PD-1) + or programmed death ligand-1 (PD-L1) + TILs;
- Type of work: reviews, case reports, conference abstracts and conference papers;
- Other conditions that did not meet the inclusion criteria.

The retrieved citations were screened by two reviewers (Yaling Wang and Yuhua Song) in terms of duplicated citations, titles, abstract sand full-texts. Only eligible trials that met the inclusion criteria were included. If there were any inconsistences, they were addressed by a discussion.

### Data abstraction

Two co-authors (Yaling Wang and Yuhua Song) independently used Microsoft Excel version 2016 (Microsoft Corporation, Redmond, Washington, USA) to collect the following information from the eligible papers: the first author, publication year, original nation, median follow-up, median age, total number of analyzed patients, the Hazard Ratio (HR) with its 95% confidence interval (CI) indicating the association of the intervention factor and OS, the event number of pCR in different intervention factor or the Odds ratio (OR) with the 95%CI referencing the association between the intervention factor and pCR, as well as the event number of the presentation of high-level TILs in different subtypes. If some divergences existed, they were resolved by the third co-author (Xuezhen Ma).

### Statistical analysis

We protocoled each 10% increment of TILs and high-level TILs in breast tumors as the study groups and non-10% increment of TILs and low-level TILs in tumors as the control groups. If the trials reported the event number of pCR in the study cohort and the control cohort, respectively, the crude OR with its 95% CI was calculated and pooled with that from the other studies. In the analysis of the impact of the intervention factors on OS, the crude HRs with their 95% CIs from the included studies were directly pooled. The comparison of the expression of high-level TILs across the three subtypes was computed in terms of the event and total numbers. If the publication was lacking the event number, it was obtained according to the incidence rate of the event or other information. The heterogeneity among analyzed trials was assessed by the heterogeneity *χ*^*2*^ test (significant level of *p* < 0.1) with its *I*^*2*^ value. The fixed-effect model was used to pool the data if the heterogeneity test of the meta-analysis was not statistically significant; otherwise, the random-effect model was utilized. The publication bias of these analyses was evaluated by the Egger’s test (significant level of *p* < 0.05). The ER status, primary endpoint, and the chemotherapy strategy as well as the chemotherapy regimen as well as the TILs subset in the eligible studies were also discussed. All the statistical tests were conducted by StataSE software version 12.0 (StataCorp LP, College Station, TX, USA).

## Results

### Search results

**Fig. 1:**
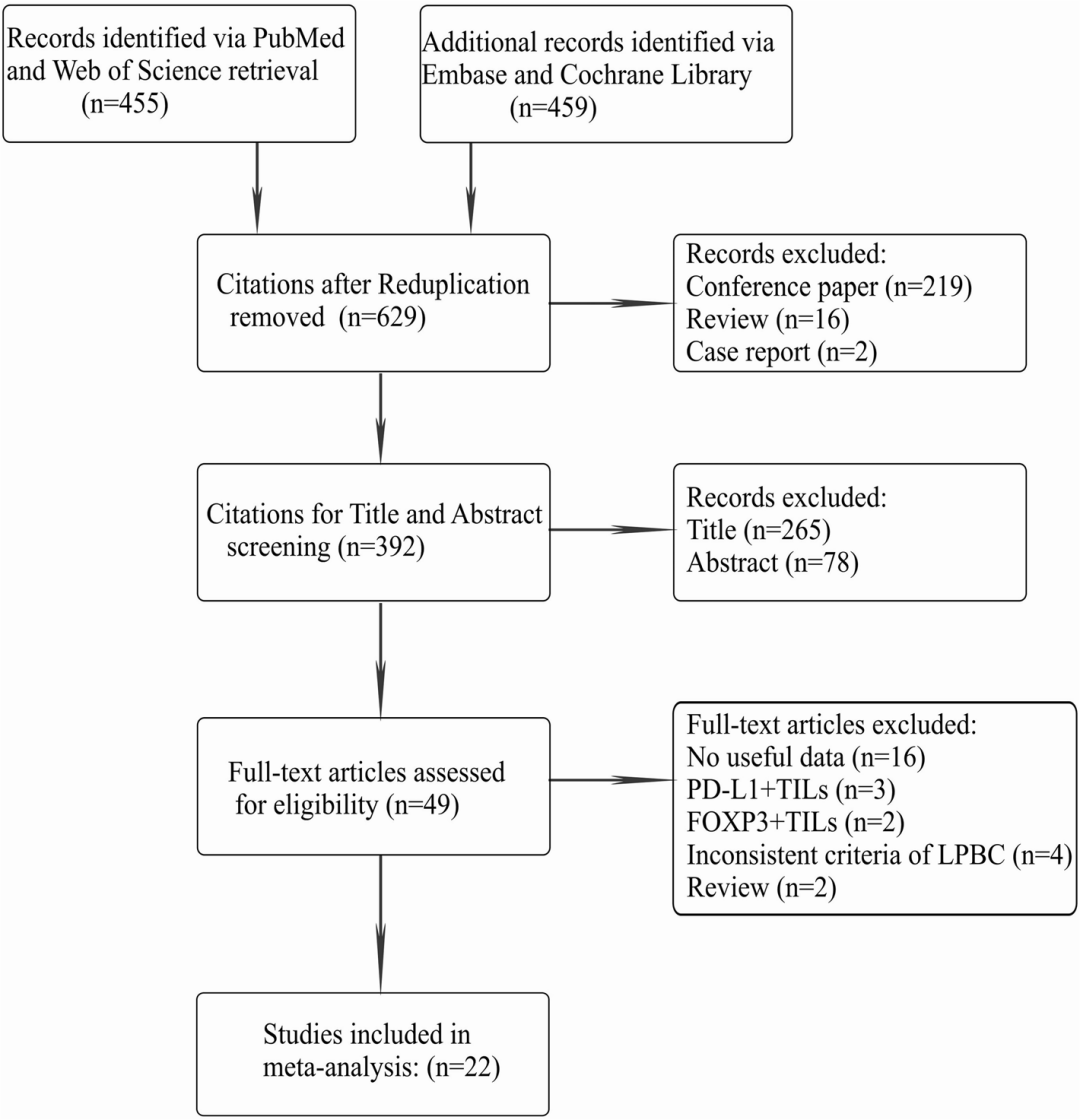
Flow diagram of eligible article selection

After the systematic retrieval from the abovementioned databases, a total of 914 initial citations were obtained by using the search strategy, and 392 potential citations were left for title and abstract screening following the deletions of duplications (*n* = 285), conference papers (*n* = 219), reviews (*n* = 16) and case reports (n = 2). Next, 49 articles remained for full-text assessment due to 343 citations being excluded via title and abstract screening; of these, studies that were reviews (*n* = 2), inconsistent to the criteria of the high-level TILs in our study (*n* = 4), devoid of useful data (*n* = 16) and centered on PD-L1 + TILs (*n* = 3) or FOXP3+ TILs (*n* = 2) did not meet the inclusion criteria and hence were excluded. Ultimately, 22 qualified studies were included for meta-analysis (Table 1) [5, 6, 8–27]. The procedure of qualified article selection is outlined in Fig. 1**.**

**Table 1:** Details of the included trials

Study (Trail)	Publication Year	Study duration	Original nation	Median follow-up	No. of patient(n)	ER status	Primary endpoints	Chemotherapy strategy	Regimen
West [8]	2011	Unknow	Canada	Unknow	111	Negative	pCR	NAC	FEC or TET
Seo [9]	2013	2004–2011	Korea	Unknow	153	Both	pCR	NAC	AC or ACT or AD
Lee [10]	2013	2000–2009	Korea	Unknow	175	Both	pCR	NAC	AC or ACT
Denkert [11]^*^	2010	1999–2001	Germany	Unknow	218	Both	pCR	NAC	ACT
Denkert [11]^*^	2010	2002–2005	Germany	Unknow	840	Both	pCR	NAC	TAC or TAC followed by vinorelbine and capecitabine
Denkert [12]	2015	Unknow	Germany	Unknow	580	Negative	pCR	Unknow	1:1 to PM or PMCb
Watanabe [13]	2018	2008–2016	Japan	26.1 m	197	Both	pCR	NAC	Anthracycline- or taxane- or anthracycline- plus taxane-based
Galvez [14]	2018	2003–2014	Peru	Unknow	435	Both	pCR	NAC	ACP or AC
Hida [15]	2016	2007–2014	Japan	Unknow	159	Negative	pCR	NAC	Unknow
Denkert [6]	2018	Unknow	Germany	Unknow	3771	Both	pCR, OS	NAC	Docetaxel- or paclitaxel- or nab-paclitaxel-based
Hwang [16]	2019	2004–2013	Korea	60.1 m	248	Both	pCR	NAC	Anthracycline- plus taxane-based
Kim [17]	2016	2004–2011	Korea	6.4y	688	Negative	OS	NAC	AC or ACT or ACP
Sønderstrup [18]	2019	1997–2011	Denmark	5.8y	399	Both	OS	Unknow	Unknow
Pruneri [19]	2016	1995–2010	Switzerland	8.2y	897	Negative	OS	AC	CMF, CMF + AC
Pruneri [20]	2016	Unknow	Italy	6.9y	647	Negative	OS	AC	1:1 to CM or no-CM
Tian [21]	2016	2008–2012	China	4y	372	Negative	OS	AC	Anthracycline- or anthracycline- plus taxanes-based
Adams [22]	2014	Unknow	USA	10.6y	481	Negative	OS	NAC	ACT or ACP
Loi [5]	2014	Unknow	Belgium	62 m	934	Both	OS	NAC	Docetaxel or vinorelbine followed by FEC
Kochi [23]	2018	Unknow	Japan	Unknow	40	Both	OS	NAC	Anthracycline- plus taxane-based
Dieci [24]	2015	Unknow	France	12.7y	781	Both	OS	AC	Anthracycline-based
Luen [25]	2019	Unknow	Australia	6y	375	Negative	OS	NAC	Unknow
Luen [26]	2017	Unknow	Australia	50 m	678	Negative	OS	AC	1:1 to trastuzumab and docetaxel plus either pertuzumab or placebo
Burugu [27]	2017	1989–2002	Canada	13y	2497	Both	Others	AC	Unknow

*This article is divided into two researches due to different regimen

*Abbreviations:* pCR, pathological complete response; OS, overall survival; NAC, neoadjuvant chemotherapy; AC, adjuvant chemotherapy; TNBC, triple-negative breast cancer; HER2 + BC, human epidermal growth factor receptor 2 positive breast cancer

*Regimen explanation:* FEC: fluorouracil, epirubicin, and cyclophosphamide; TET: docetaxel followed by epirubicin plus docetaxel; AC: doxorubicin and cyclophosphamide; ACT: AC followed by docetaxel; AD: doxorubicin and docetaxel; TAC: docetaxel, doxorubicin, and cyclophosphamide; PM: Paclitaxel and non-pegylated liposomal doxorubicin; PMCb: Paclitaxel and non-pegylated liposomal doxorubicin followed by carboplatin; TP: paclitaxel plus platinum; ACP: doxorubicin and cyclophosphamide followed by paclitaxel; CAF: cyclophosphamide, adriamycin and fluorouracil; CMF: cyclophosphamide, methotrexate and fluorouracil; CM: cyclophosphamide plus methotrexate

Of those included studies, the publication year ranged from 2010 to 2019, 14 (63.6%) were retrospective studies with a total of 6958 cases, 10 (45.5%) were originally from Asian countries, 9 (40.9%) documented the breast cancer patients with ER-negative status, and the predominately chemotherapy strategy was in the setting of neoadjuvant therapy. Table 2 additionally represented the other details involving the median follow-up, publication year, the median age, the analyzed cases in each analysis, the primary endpoint, and the detailed chemotherapy regimen, as well as the TILs subsets.

**Table 2:** Summary of the characteristics of the 21 included Studies

Characteristic	Studies, No. (%) (***N*** = 22)	Primary Breast Cancer Patients, No. (%) (***N*** = 15,676)
Study type		
Randomized trial	5 (22.7)	3578 (22.8)
Retrospective	14 (63.6)	6958 (44.4)
Pooled	1 (4.5)	3771 (24.1)
Prospective–retrospective	1 (4.5)	934 (6.0)
Prospective	1 (4.5)	435 (2.8)
Publication date, median (range), y	2016 (2010–2019)	
Follow-up, median (range), mo^*^	90.6 (48.0–190.8)	
Median age, median (range), y^*^	50.0 (46.5–54.0)	
10% increment of TILs and OS, total (range), n		
All subtypes	4460 (399–2346)	
Luminal	1886 (463–832)	
HER2-enriched	1985 (112–986)	
TNBC	3847 (92–897)	
10% increment of TILs and pCR, total (range), n	1638 (218–840)	
LPBC and pCR, total (range), n		
All subtypes	6697 (40–3771)	
Luminal	1717 (91–1366)	
HER2-enriched	1801 (40–1379)	
TNBC	1425 (48–906)	
High TILs across different subtypes, total (range), n		
TNBC vs Luminal	6524 (138–2297)	
HER2-enriched vs Luminal	6696 (149–2745)	
TNBC vs HER2-enriched	3722 (105–2285)	
Original area		
Asia	10 (45.5)	3085 (19.7)
America	4 (18.2)	3524 (22.5)
Europe	8 (36.4)	9067 (57.8)
ER status		
ER-positive	0 (0.0)	0 (0.0)
ER-negative	9 (40.9)	4300 (27.4)
ER-both	13 (59.1)	11,376 (72.6)
Primary endpoint		
pCR	10 (45.5)	6834 (43.6)
OS	11 (50.0)	6345 (40.5)
Others	1 (4.5)	2497 (15.9)
Chemotherapy strategy		
Neoadjuvant	15 (68.2)	9669 (61.7)
Adjuvant	5 (22.7)	5028 (32.1)
Unknow	2 (9.1)	979 (6.2)
Chemotherapy regimen		
Anthracycline-based	3 (13.6)	2113 (13.5)
Taxanes-based	1 (4.5)	3771 (24.1)
Anthracycline- and taxanes-based	10 (45.5)	3822 (24.4)
Methotrexate-based	3 (13.6)	1915 (12.2)
Unknow	5 (22.7)	4055 (25.9)
TILs subsets		
TILs	11 (50.0)	8014 (51.1)
iTILs	3 (13.6)	4135 (26.4)
sTILs	6 (27.3)	3199 (20.4)
CD8 + TILs	1 (4.5)	175 (1.1)
CD4 + TILs	1 (4.5)	153 (1.0)

Abbreviations: TILs, tumor-infiltrating lymphocytes; OS, overall survival; HER2, human epidermal growth factor receptor 2; TNBC, triple-negative breast cancer; pCR, pathological complete response; LPBC, lymphocyte-predominant breast cancer; ER, estrogen receptor; iTILs, intratumoral tumor-infiltrating lymphocytes; sTILs stromal tumor-infiltrating lymphocytes

*Median value is calculated in terms of available data

### Association of each 10% increment of TILs and OS

**Fig. 2:**
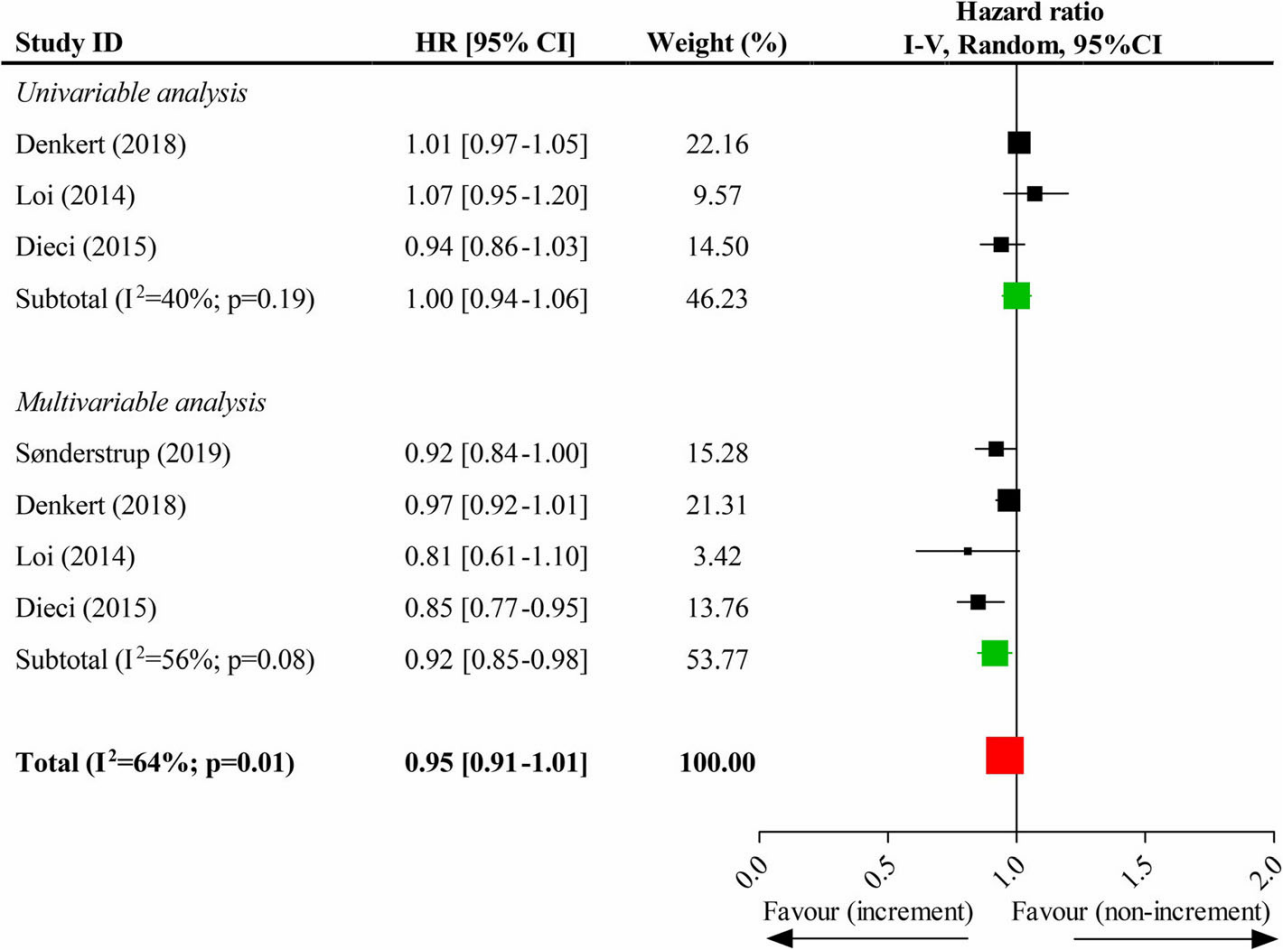
Impacts of each 10% increment of tumor-infiltrating lymphocytes in breast tumor on overall survival

Four studies recorded each 10% increment of TILs and OS in breast cancers without classification to different molecular subtypes, and the pooled results suggested that each 10% increment of TILs could not significantly improve OS (HR, 0.95; 95% CI, 0.91–1.01). However, there was a significant improvement in OS in terms of the pooled results of multivariate data (HR, 0.92; 95% CI, 0.85–0.98) but not that of univariate data (HR, 1.00; 95% CI, 0.94–1.06) (Fig. 2). In the subgroup analysis of different subtypes, the pooled results showed that, although each 10% increment of TILs in luminal tumor phenotype did not significantly improve OS (HR, 1.06; 95% CI, 0.99–1.13) (**eFigure 1**, *Supplementary page 1*), the improvements in OS were attained by it in HER2-overexpression (HR, 0.92; 95% CI, 0.89–0.95) (**eFigure 2**, *Supplementary page 1*) and TN (HR, 0.90; 95% CI, 0.89–0.92) subtypes (**eFigure 3**, *Supplementary page 2*). The results were both statistically significant in pooling the univariate data and the multivariate data of the latter two molecular phenotypes (these data were shown in **eFigure2** and **eFigure 3,** respectively).

### Association of each 10% increment of TILs and pCR

**Fig. 3:**
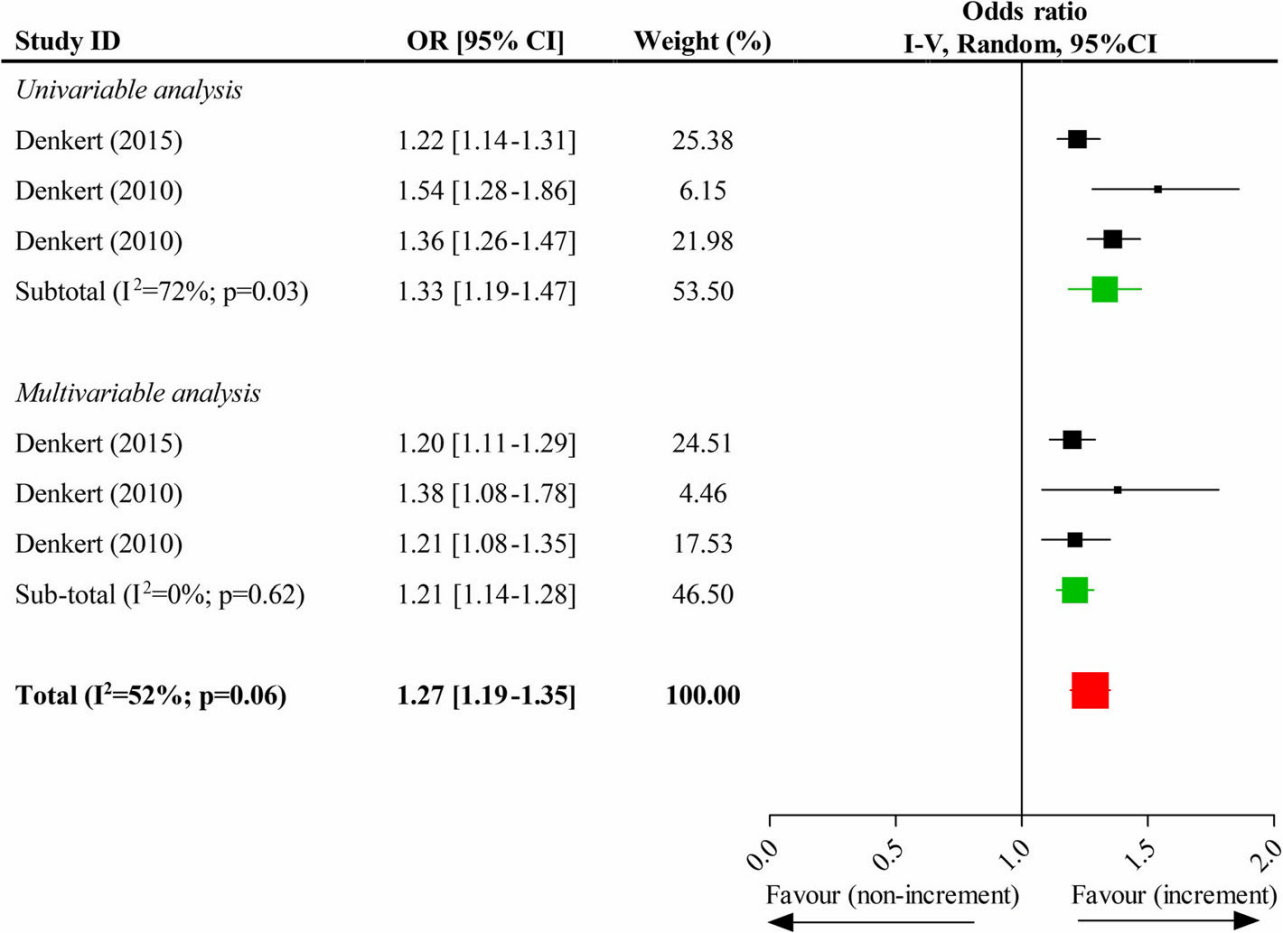
Impacts of each 10% increment of tumor-infiltrating lymphocytes in breast tumor on the pathological complete response

Two studies reported each 10% increment of TILs and pCR in breast tumors, and one [11] of them divided patients into the training cohort and the validation cohort. Thus, three independently relevant data existed. The pooled results indicated that there was a significantly positive correlation between each 10% increment of TILs and the increased pCR rate (OR, 1.27; 95% CI, 1.19–1.35). The results of pooling univariate data (OR, 1.33; 95% CI, 1.19–1.47) and multivariate data (OR, 1.21; 95% CI, 1.14–1.28) were still statistically significant (Fig. 3).

### Association of high-level TILs and pCR

**Fig. 4:**
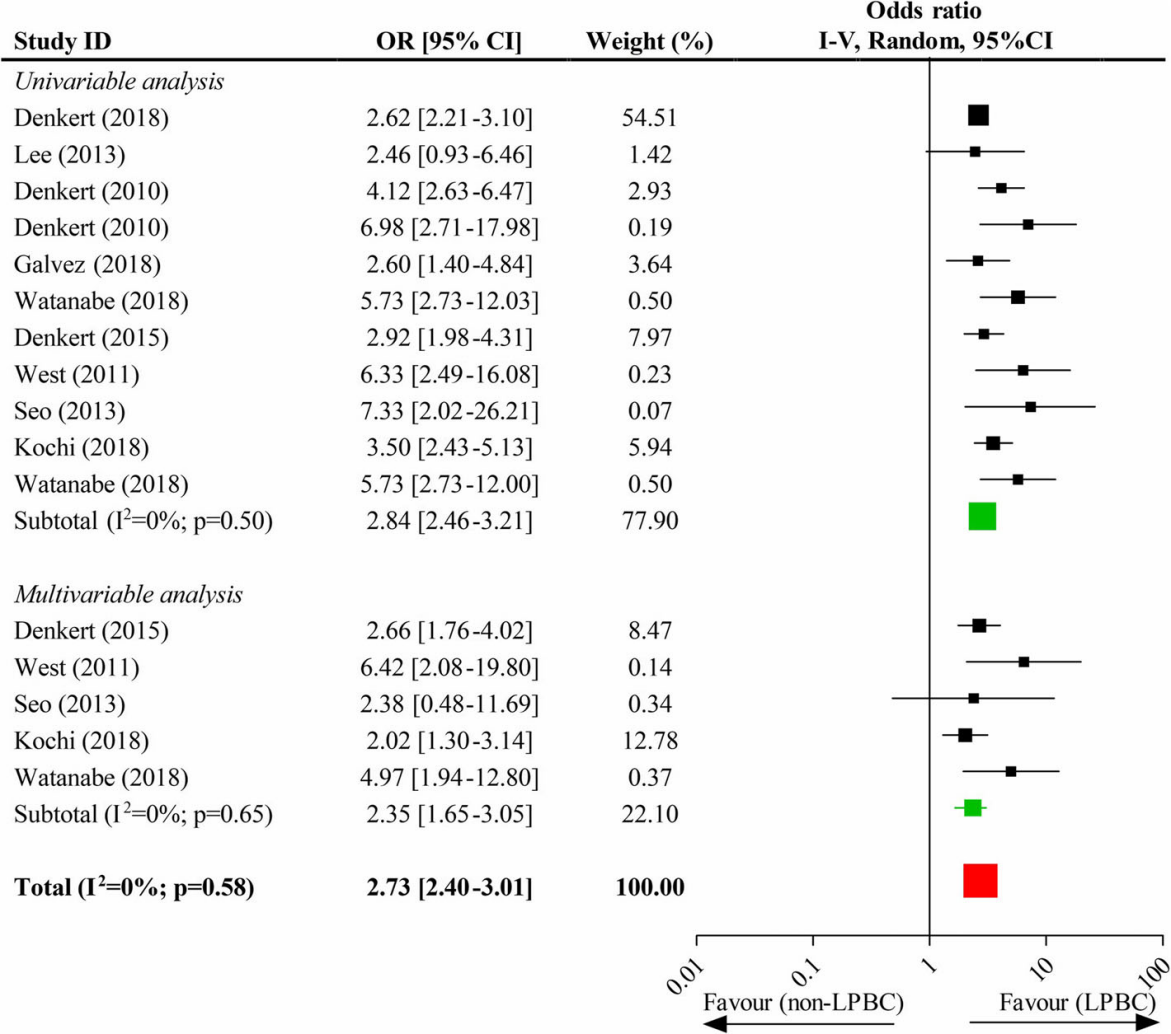
Impacts of the high-level TILs on the pathological completed response

Eleven studies provided sufficient data to the association of high-level TILs and pCR. There was a significant difference in pCR rate between high-level and low-level TILs (OR, 2.73; 95% CI, 2.40–3.01), and the pooled results of univariate data (OR, 2.84; 95% CI, 2.46–3.21) and multivariate data (OR, 2.35; 95% CI, 1.65–3.05) were also both statistically significant (Fig. 4). In the subgroup analysis, the pooled results all indicated a higher pCR rate in luminal, HER2-overexpression and TN phenotypes with high-level TILs than those with low-level TILs, respectively (these data were outlined in **eFigure 4**, *Supplementary page 3*).

### Comparison of high-level TILs expression across different breast cancer subsets

**Fig. 5:**
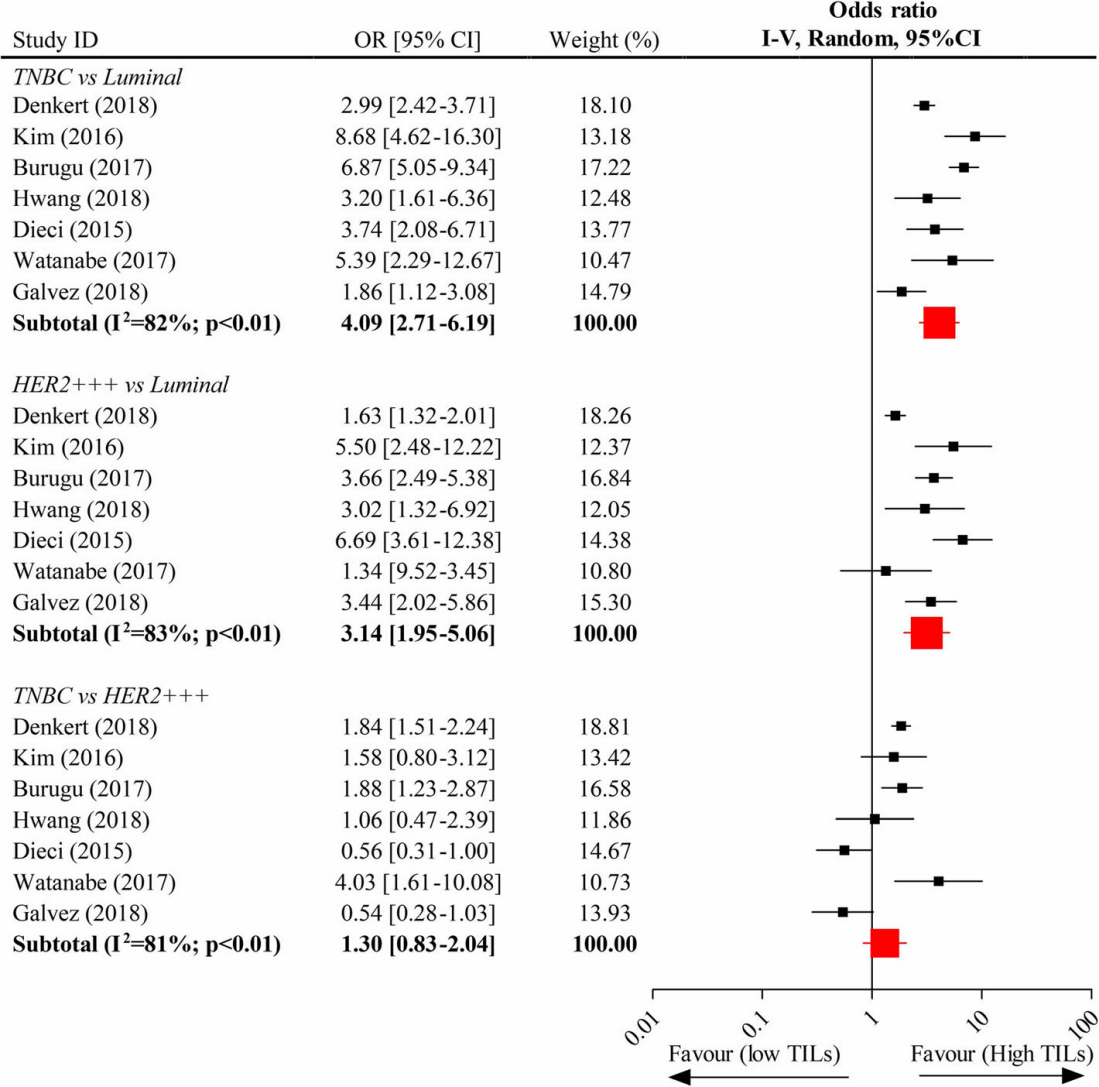
Comparison of the expression of high-level TILs across different subtypes of breast tumors

Seven studies were collected to perform the comparison of expression of high-level TILs across the different subsets of breast tumors. The pooled data of analysis showed that the presentation of high-level TILs between HER2-overexpression subtype and TN subtype was not significantly different (OR, 1.30; 95%CI, 0.83–2.04), whereas both subtypes experienced a significantly elevated expression of high-level TILs as compared to luminal phenotype (HER2-overexpression vs. luminal, OR, 3.14; 95% CI, 1.95–5.06; and TN vs. luminal, OR, 4.09; 95% CI, 2.71–6.19; respectively) (Fig. 5).

### Publication bias

Several meta-analyses manifested moderate-to-considerable heterogeneity, and therefore, the random-effect model was employed to pool the data. With the exception of the impact of each 10% increment of TILs in TN tumor subtype on OS (*p* = 0.001) and that of the high-level TILs on pCR (*p* = 0.007), there was no likelihood of publication bias in others because the Egger’s tests of them were not statistically significant (**eTable 1**, *Supplementary page 4)*. The funnel plots for both analyses with significant publication bias were presented in **eFigure 5** *(Supplementary page 5)*.

## Discussion

Previous meta-analyses have shown that the value of total TILs is that they are associated with an improved outcome in breast cancer following neoadjuvant chemotherapy but not in ER-negative subtypes [2]. The prognostic and predictive importance of TILs in ER+ disease is still controversial. To investigate this issue, we evaluate all available evidence regarding ER positive and ER-negative breast tumors from a pool of clinical studies and demonstrate that each 10% increment of TILs in breast tumors improves OS in HER2-amplified and TN molecular subtypes but not in the luminal phenotype.

Our results also agree with Denkert’s [6] and West’s trials, [8] which both suggest that a high TILs concentration increases the tumor response to neoadjuvant chemotherapy and anthracycline-based chemotherapy, and is in association with better long-term survival in the HER2-overexpression and TN breast tumors. A pooled analysis identified an appropriate cut-off of sTILs for early-stage, node-negative TN breast cancer patients, in which those patients with sTILs≥30% who underwent adjuvant treatment benefited the excellent disease-free survival and OS [28]. Similarly, our meta-analysis confirms a suitable cut-off of TILs≥50% for all molecular subtypes of primary breast tumors because the high-level TILs predict a better pCR than the low-level TILs. Prospective de-escalation clinical trials in HER2+ and TNBC are needed to identify the appropriate TIL-levels to safely de-escalate therapy in those patients that have an excellent outcome.

In the study by Denkert et al., [6] it is found that the increased TILs may be an adverse factor to OS in breast cancer patients with the luminal subtype, which differs from our results. This difference may be explained as follows. First, they only evaluated the OS in luminal-HER2-negative tumors, while our study also includes luminal-HER-positive of breast cancer patients. Furthermore, they only center on the assessment of the impact of sTILs on OS, but we additionally assess the iTILs. Last, the treatment strategies are not identical, as only neoadjuvant chemotherapy is included in their study but adjuvant chemotherapy is yet included in ours. Collectively, the prognostic outcome of TIL-levels in luminal disease remains an important area of investigation. One of the reasons why TIL-counts are not associated with survival benefit in luminal disease is that the range of TILs is not very high, with most cases having less than 10% stromal TILs, so most series don’t have a significant number of patients with luminal disease with very high TILs. In luminal disease, combined features of immunity and tumoral characteristics, like for example tumor cell proliferation or histological grade, may be probably the best approach, while in TNBC and HER2+ disease immunity is probably sufficient to predict outcome, with cancer cell characteristics being less important in these subtypes.

A large number of clinical trials are enthusiastic about the association between the presence of TILs and the pCR rate after chemotherapy in ER-negative breast cancers. In the 2013 San Antonio Breast Cancer Symposium, Loi and colleagues [5] reported that the presentation of TILs was associated with a higher pCR rate in the HER2-overexpression and TN phenotypes of breast tumors that underwent neoadjuvant chemotherapy (HER2-overexpression breast cancer routinely received trastuzumab treatment). These results map to our findings that each 10% increment of TILs preages a greater pCR rate in breast carcinoma, but it is imperfect as lack of enough data to perform the subgroup analysis of different disease subtypes. Consequently, the understudied association between each 10% increment of TILs and pCR rate in luminal breast cancer needs to be warranted. Of note, the association between different molecular subtypes of the high-level TILs and pCR rate is well delineated in our study, i.e. the increased pCR rate favors all subtypes with the high-level TILs when compared to those with the low-level TILs. Consistently, West and colleagues reaffirm that HER2-amplified and TN subtypes with the high-level TILs have a promising chemosensitivity to anthracycline-based adjuvant or neoadjuvant chemotherapy [8].

The limitations of this study are as follows: (i) Selection bias might exist because of the inclusion criterion that limited the condition to English publications and the exclusion criteria that omitted the immune cell subsets of PD-1 + TILs, PD-L1 + TILs and FOXP3 + TILs. (ii) To obtain more evidence and larger scale of cases, analysis of pCR rate between the high-level and the low-level TILs also included trials centering on CD8 + LPBC, giving rise to considerable heterogeneity. (iii) There may be clinical heterogeneity among the included studies, such as application of different chemotherapy regimens and treatment strategies, as well as investigation of different TILs subtypes.

Despite these limitations, this was the first meta-analysis that systematically evaluated the influence of each 10% increment of TILs and the high-level TILs in breast cancer on OS and pCR, and compared the presentation of high-level TILs across different molecular subtypes. Future studies will need to supplement the underrecognized and understudied landscapes whether a higher pCR rate is related to each 10% increment of TILs in the luminal subtype of breast cancer and the high-level TILs in clinical high-risk luminal breast cancer patients can translate into a promising OS.

## Conclusions

Each 10% increment of TILs in breast tumors predicts improved OS and pCR rate of patients, specifically in the HER2-overexpression and TN molecular subtypes. Moreover, all subsets with the high-level TILs benefit greater pCR rate than those with the low-level TILs. Although there is no difference between the expression of high-level TILs among HER2-overexpression and TN phenotypes of breast cancer, they both have greater expression than that relative to the luminal tumor subtype.

## Supplementary information

**Additional file 1: eFigure 1.** Impacts of each 10% increment of tumor-infiltrating lymphocytes on overall survival in Luminal subtype. eFigure 2: Impacts of each 10% increment of tumor-infiltrating lymphocytes on overall survival in HER2-overexpression subtype. eFigure 3: Impacts of each 10% increment of tumor-infiltrating lymphocytes on overall survival in triple-negative subtype. eFigure 4: Impacts of the high-level TILs on the pathological completed response in different tumor subtypes. eTable 1. Publication bias by Egg’s test in meta-analysis. eFigure 5. Funnel plots for the significant analyses of publication bias.

## Data Availability

Not applicable.

## Abbreviations

TILs: Tumor-infiltrating lymphocytes
LPBC: Lymphocyte-predominant breast cancer
HER2: Human epidermal growth factor receptor 2
TN: Triple-negative
DFS: Disease-free survival
OS: Overall survival
pCR: Pathologic complete response
iTILs: Intratumoral TILs
sTILs: Stromal TILs
PD-1: Programmed death 1
PD-L1: Programmed death ligand-1
FOXP3: Forkhead box P3
HR: Hazard Ratio
CI: Confidence interval
OR: Odds ratio
ER: Estrogen receptor

## References

[1] Liotta LA, Kohn EC (2001). The microenvironment of the tumour-host interface. Nature..

[2] Yu X (2016). Zhang Z. Prognostic and predictive value of tumor-infiltrating lymphocytes in breast cancer: a systematic review and meta-analysis.

[3] Swann JB, Smyth MJ (2007). Immune surveillance of tumors. J Clin Invest.

[4] Loi S, Sirtaine N, Piette F, Salgado R, Viale G, Van Eenoo F (2013). Prognostic and predictive value of tumor-infiltrating lymphocytes in a phase III randomized adjuvant breast cancer trial in node-positive breast cancer comparing the addition of docetaxel to doxorubicin with doxorubicin-based chemotherapy: BIG 02-98. Journal of clinical oncology : official journal of the American Society of Clinical Oncology..

[5] Loi S, Michiels S, Salgado R, Sirtaine N, Jose V, Fumagalli D (2014). Tumor infiltrating lymphocytes are prognostic in triple negative breast cancer and predictive for trastuzumab benefit in early breast cancer: results from the FinHER trial. Annals of oncology : official journal of the European Society for Medical Oncology..

[6] Denkert C, von Minckwitz G, Darb-Esfahani S, Lederer B, Heppner BI, Weber KE (2018). Tumour-infiltrating lymphocytes and prognosis in different subtypes of breast cancer: a pooled analysis of 3771 patients treated with neoadjuvant therapy. The Lancet Oncology..

[7] Salgado R, Denkert C, Demaria S, Sirtaine N, Klauschen F, Pruneri G (2015). The evaluation of tumor-infiltrating lymphocytes (TILs) in breast cancer: recommendations by an international TILs working group 2014. Annals of oncology : official journal of the European Society for Medical Oncology.

[8] West NR, Milne K, Truong PT, Macpherson N, Nelson BH, Watson PH. Tumor-infiltrating lymphocytes predict response to anthracycline-based chemotherapy in estrogen receptor-negative breast cancer. Breast Cancer Research. 2011;13(6). 10.1186/bcr3072 PMC3326568 22151962

[9] Seo AN, Lee HJ, Kim EJ, Kim HJ, Jang MH, Lee HE (2013). Tumour-infiltrating CD8+ lymphocytes as an independent predictive factor for pathological complete response to primary systemic therapy in breast cancer. Br J Cancer.

[10] Lee HJ, Seo JY, Ahn JH, Ahn SH, Gong G (2013). Tumor-associated lymphocytes predict response to Neoadjuvant chemotherapy in breast Cancer patients. J Breast Cancer.

[11] Denkert C, Loibl S, Noske A, Roller M (2010). M¨^1^ller BM, Komor M, et al. tumor-associated lymphocytes as an independent predictor of response to neoadjuvant chemotherapy in b reast cancer. Journal of clinical oncology : official journal of the American Society of Clinical Oncology.

[12] Denkert C, von Minckwitz G, Brase JC, Sinn BV, Gade S, Kronenwett R (2015). Tumor-infiltrating lymphocytes and response to neoadjuvant chemotherapy with or without carboplatin i n human epidermal growth factor receptor 2-positive and triple-negative primary breast cancers. Journal of clinical oncology : official journal of the American Society of Clinical Oncology..

[13] Watanabe T, Hida AI, Inoue N, Imamura M, Fujimoto Y, Akazawa K (2018). Abundant tumor infiltrating lymphocytes after primary systemic chemotherapy predicts poor prognosis in estrogen receptor-positive/HER2-negative breast cancers. Breast Cancer Res Treat.

[14] Galvez M, Castaneda CA, Sanchez J, Castillo M, Rebaza LP, Calderon G (2018). Clinicopathological predictors of long-term benefit in breast cancer treated with neoadjuvant chemotherapy. World Journal Of Clinical Oncology.

[15] Hida AI, Sagara Y, Yotsumoto D, Kanemitsu S, Kawano J, Baba S (2016). Prognostic and predictive impacts of tumor-infiltrating lymphocytes differ between triple-negative and HER2-positive breast cancers treated with standard systemic therapies. Breast Cancer Res Treat.

[16] Hwang HW, Jung H, Hyeon J, Park YH, Ahn JS, Im YH (2019). A nomogram to predict pathologic complete response (pCR) and the value of tumor-infiltrating lymphocy tes (TILs) for prediction of response to neoadjuvant chemotherapy (NAC) in breast cancer patients. Breast Cancer Res Treat.

[17] Kim YA, Lee HJ, Heo SH, Park HS, Park SY, Bang WS (2016). MxA expression is associated with tumor-infiltrating lymphocytes and is a prognostic factor in triple-negative breast cancer. Breast Cancer Res Treat.

[18] Sonderstrup IMH, Jensen MB, Ejlertsen B, Eriksen JO, Gerdes AM, Kruse TA, et al. Evaluation of tumor-infiltrating lymphocytes and association with prognosis in BRCA-mutated breast cancer. Acta oncologica (Stockholm, Sweden). 2019:1–8. 10.1080/0284186X.2018.1539239 30614364

[19] Pruneri G, Gray KP, Vingiani A, Viale G, Curigliano G, Criscitiello C (2016). Tumor-infiltrating lymphocytes (TILs) are a powerful prognostic marker in patients with triple-negative breast cancer enrolled in the IBCSG phase III randomized clinical trial 22-00. Breast Cancer Res Treat.

[20] Pruneri G, Vingiani A, Bagnardi V, Rotmensz N, De Rose A, Palazzo A (2016). Clinical validity of tumor-infiltrating lymphocytes analysis in patients with triple-negative breast cancer. Ann Oncol.

[21] Tian T, Ruan M, Yang W, Shui R (2016). Evaluation of the prognostic value of tumor-infiltrating lymphocytes in triple-negative breast cancers. Oncotarget..

[22] Adams S, Gray RJ, Demaria S, Goldstein L, Perez EA, Shulman LN (2014). Prognostic value of tumor-infiltrating lymphocytes in triple-negative breast cancers from two phase I II randomized adjuvant breast cancer trials: ECOG 2197 and ECOG 1199. Journal of clinical oncology : official journal of the American Society of Clinical Oncology..

[23] Kochi M, Iwamoto T, Niikura N, Bianchini G, Masuda S, Mizoo T (2018). Tumour-infiltrating lymphocytes (TILs)-related genomic signature predicts chemotherapy response in breast cancer. Breast Cancer Res Treat.

[24] Dieci MV, Mathieu MC, Guarneri V, Conte P, Delaloge S, Andre F (2015). Prognostic and predictive value of tumor-infiltrating lymphocytes in two phase III randomized adjuvant breast cancer trials. Ann Oncol.

[25] Luen SJ, Salgado R, Dieci MV, Vingiani A, Curigliano G, Gould RE (2019). Prognostic implications of residual disease tumor-infiltrating lymphocytes and residual cancer burden in triple-negative breast cancer patients after neoadjuvant chemotherapy. Annals of oncology : official journal of the European Society for Medical Oncology..

[26] Luen SJ, Salgado R, Fox S, Savas P, Eng-Wong J, Clark E (2017). Tumour-infiltrating lymphocytes in advanced HER2-positive breast cancer treated with pertuzumab or placebo in addition to trastuzumab and docetaxel: a retrospective analysis of the CLEOPATRA study. The Lancet Oncology.

[27] Burugu S, Gao D, Leung S, Chia SK, Nielsen TO (2017). LAG-3+tumor infiltrating lymphocytes in breast cancer: clinical correlates and association with PD-1/PD-L1+tumors. Ann Oncol.

[28] Loi S, Drubay D, Adams S, Pruneri G, Francis PA, Lacroix-Triki M (2019). Tumor-infiltrating lymphocytes and prognosis: a pooled individual patient analysis of early-stage tri ple-negative breast cancers. J Clin Oncol.

