# Supplementary material for: Association between levels of tumor-infiltrating lymphocytes in different subtypes of primary breast tumors and prognostic outcomes: a meta-analysis
Source: BMC Womens Health. 2020 Sep 5;20:194. doi: 10.1186/s12905-020-01038-x (PMC7487965; doi:10.1186/s12905-020-01038-x)
Supplement: Supplementary file 1 — Additional file 1: eFigure 1. Impacts of each 10% increment of tumor-infiltrating lymphocytes on overall survival in Luminal subtype. eFigure 2: Impacts of each 10% increment of tumor-infiltrating lymphocytes on overall survival in HER2-overexpression subtype. eFigure 3: Impacts of each 10% increment of tumor-infiltrating lymphocytes on overall survival in triple-negative subtype. eFigure 4: Impacts of the high-level TILs on the pathological completed response in different tumor subtypes. eTable 1. Publication bias by Egg’s test in meta-analysis. eFigure 5. Funnel plots for the significant analyses of publication bias. [file 12905_2020_1038_MOESM1_ESM.docx]

**eFigure 1: Impacts of each 10% increment of tumor-infiltrating lymphocytes on overall survival in Luminal subtype.**


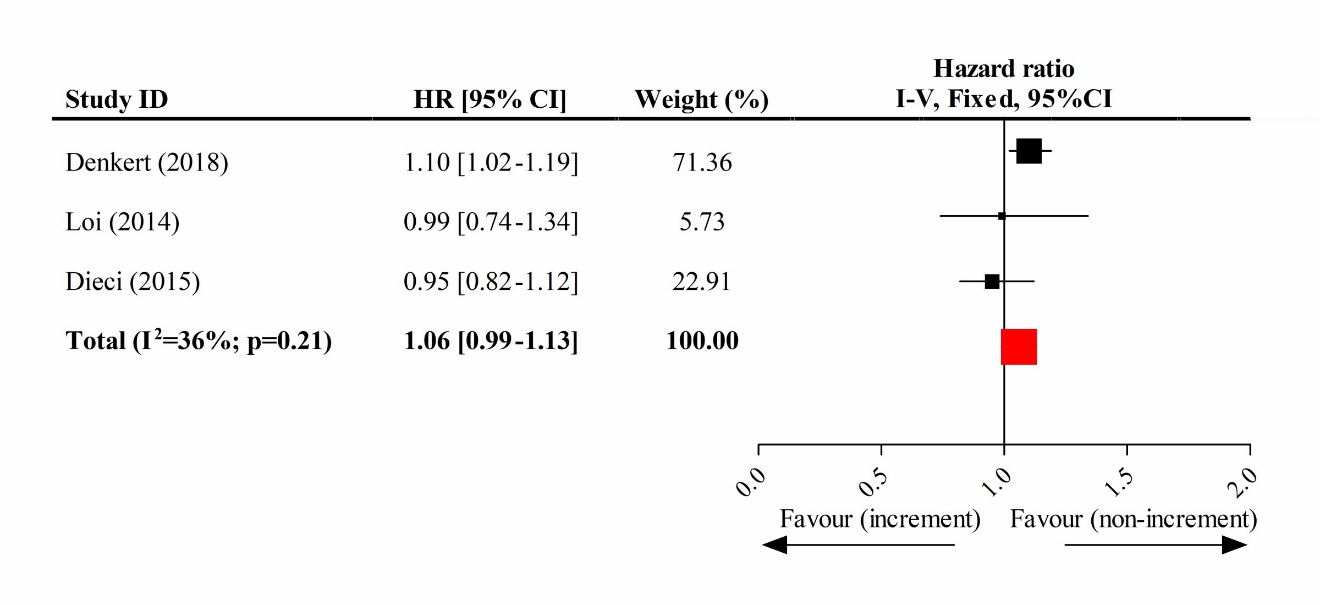


**eFigure 2: Impacts of each 10% increment of tumor-infiltrating lymphocytes on overall survival in HER2-overexpression subtype.**


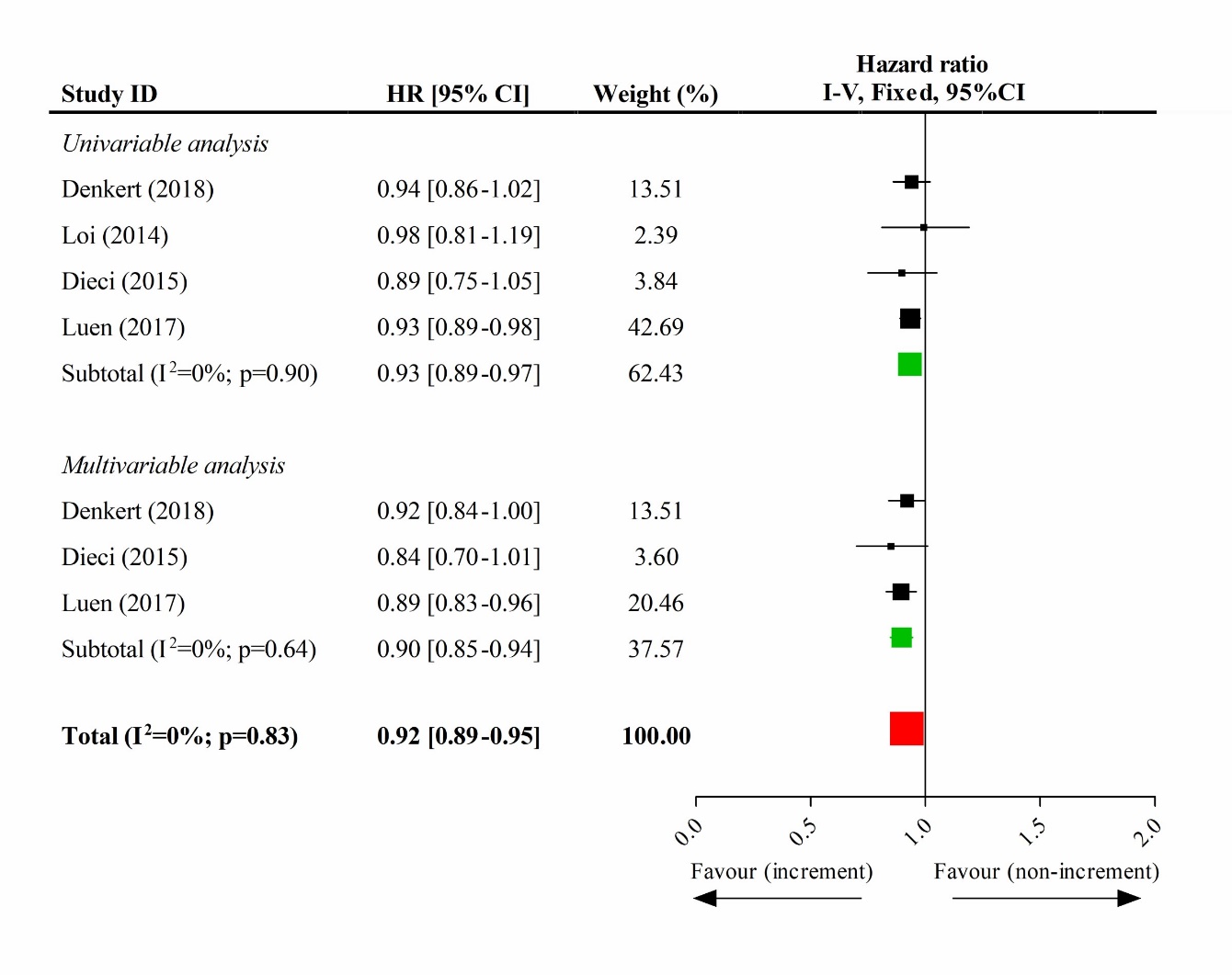


**eFigure 3: Impacts of each 10% increment of tumor-infiltrating lymphocytes on overall survival in triple-negative subtype.**


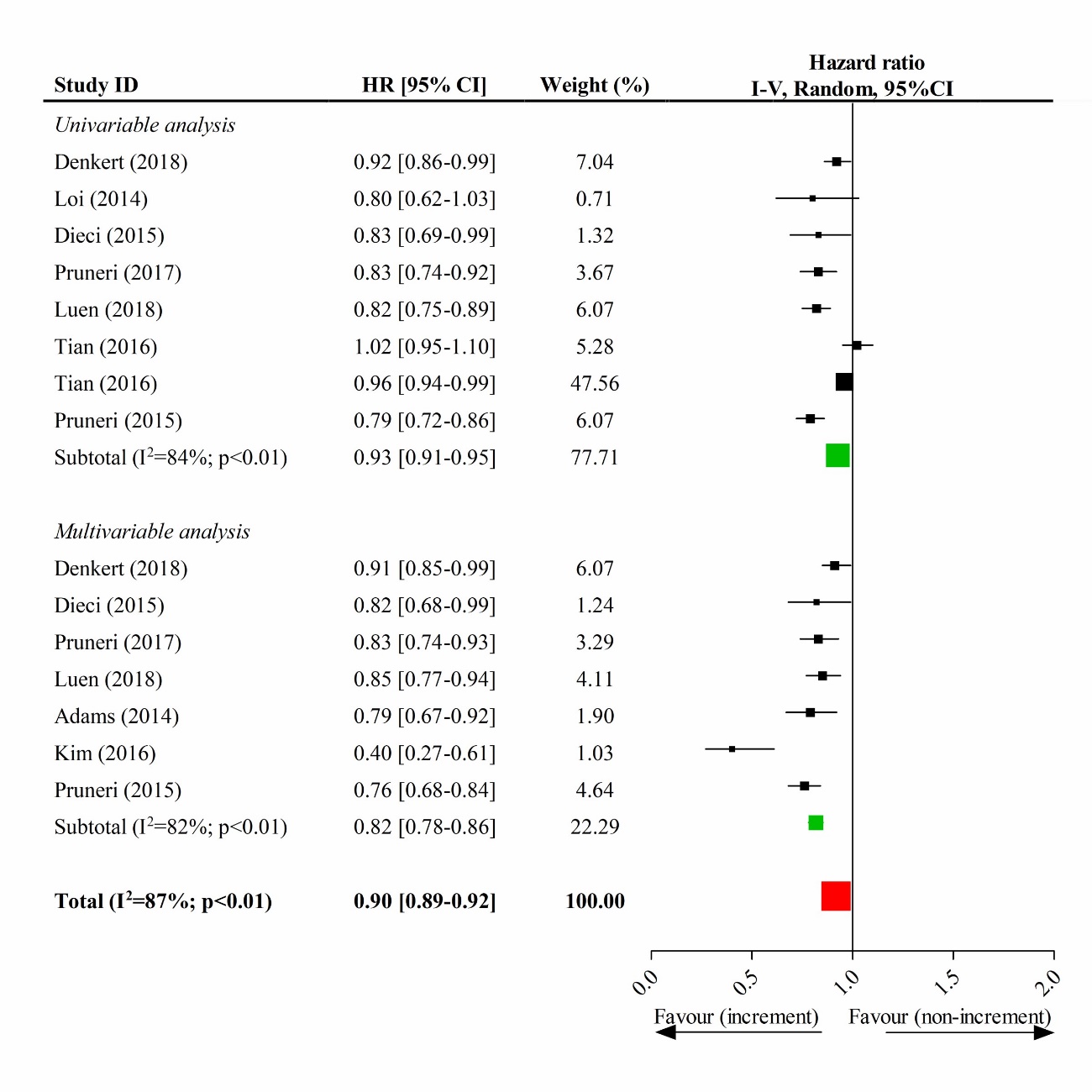


**eFigure 4: Impacts of the high-level TILs on the pathological completed response in different tumor subtypes.**


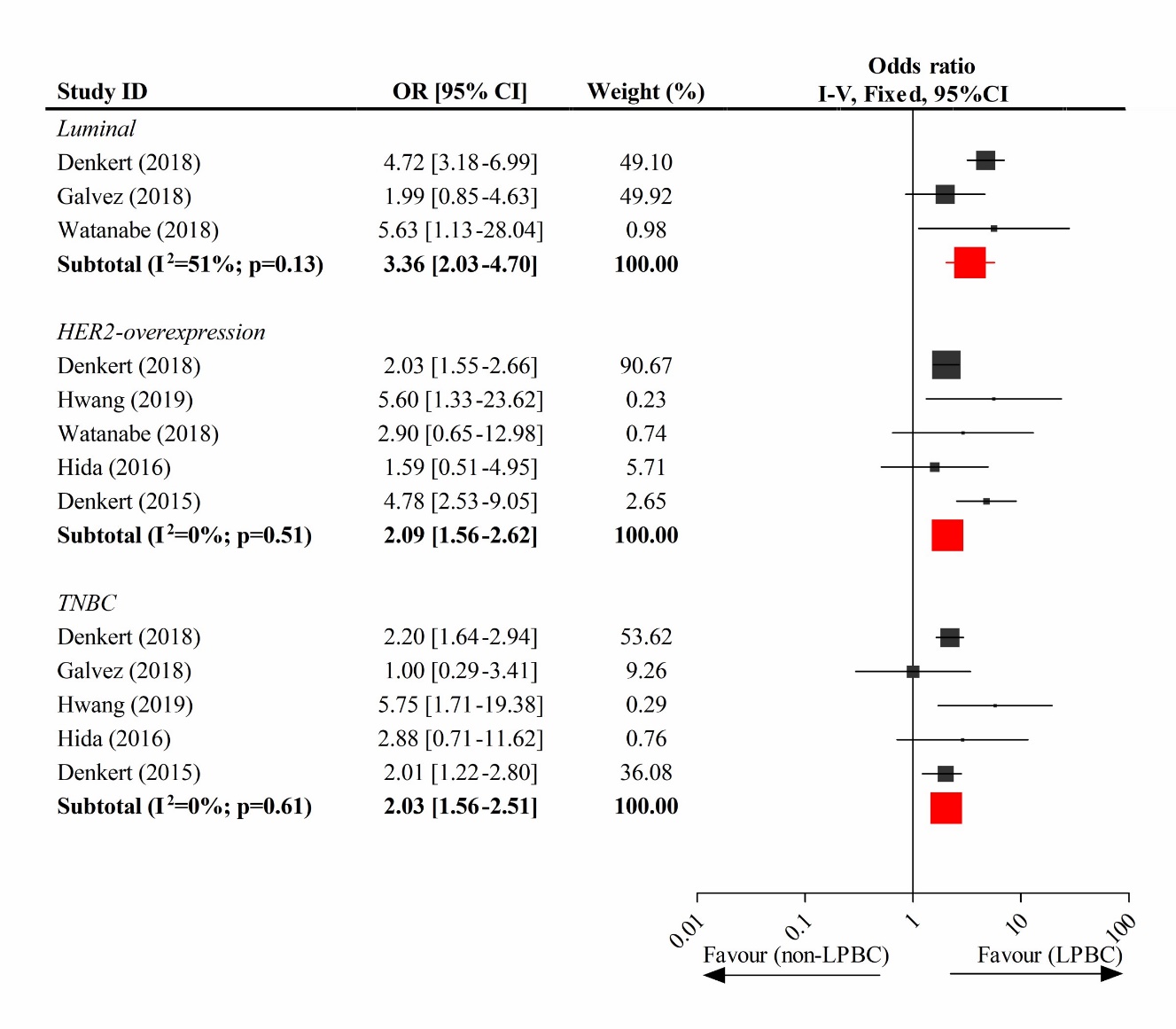


**eTable 1. Publication bias by Egg’s test in meta-analysis.**

| **Analysis label** | **p value^*^** |
| --- | --- |
| *10% increment of TILs* |  |
| OS in all subtype | 0.192 |
| OS in Luminal subtype | 0.437 |
| OS in HER2-enriched subtype | 0.536 |
| OS in TN subtype | 0.001 |
| pCR in all subtype | 0.303 |
| *High-level TILs* |  |
| pCR in all subtype | 0.007 |
| pCR in Luminal subtype | 0.745 |
| pCR in HER2-enriched subtype | 0.379 |
| pCR in TN subtype | 0.775 |
| *High-level TILs* |  |
| HER2-enriched vs Luminal | 0.115 |
| TNBC vs Luminal | 0.680 |
| TNBC vs HER2-enriched | 0.350 |

*significant level p<0.1.

Abbreviations: TILs, tumor-infiltrating lymphocytes; OS, overall survival; pCR, pathological complete response; HER2, human epidermal growth factor receptor 2; TN, triple-negative.

**eFigure 5. Funnel plots for the significant analyses of publication bias.**


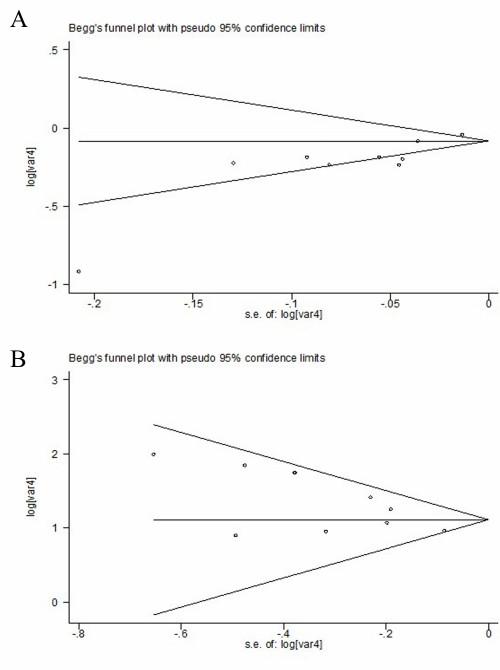


A. 10% increment of TILs and OS in TN subtype; B. High-level TILs and pCR in all subtypes
